# Supplementary material for: Validation of a Harmonised, Three-Item Cognitive Screening Instrument for the Survey of Health, Ageing and Retirement in Europe (SHARE-Cog)
Source: Int J Environ Res Public Health. 2023 Sep 30;20(19):6869. doi: 10.3390/ijerph20196869 (PMC10572728; doi:10.3390/ijerph20196869)
Supplement: Supplementary file 1 [file ijerph-20-06869-s001.zip › SHARE-Cog questionnaire.pdf]

SHARE-Cog assessment

Name:_______________________ Sex:____________ Date:____________

Date of birth _________________ Education:______________ Note:_________________________

WORD REGISTRATION

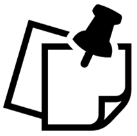

"Now, I am going to read a list of words. We have purposely made the list long so it will be difficult for anyone to recall all the words. Most people recall just a few. Please listen carefully, as the set of words cannot be repeated. When I have finished, I will ask you to recall aloud as many of the words as you can, in any order. Is this clear?"

- Select 1 of the 4 lists

"Ready?" [Read out one of the four lists below]

"Now please tell me all the words you can recall."

- Allow up to one minute for recall

SCORE AS 1 POINT PER WORD CORRECT.

TOTAL SCORE:

/10

Word list option 1	tick	Word list option 2	tick	Word list option 3	tick	Word list option 4	tick
Hotel		Sky		Woman		Water	
River		Ocean		Rock		Church	
Tree		Flag		Blood		Doctor	
Skin		Dollar		Corner		Palace	
Gold		Wife		Shoes		Fire	
Market		Machine		Letter		Garden	
Paper		Home		Girl		Sea	
Child		Earth		House		Village	
King		College		Valley		Baby	
Book		Butter		Engine		Table	

VERBAL FLUENCY

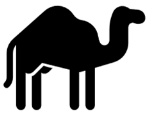

"Now I would like you to name as many different animals as you can think of. You have one minute to do this. Ready, go."

- Allow one minute precisely. If the respondent stops before the end of the time, encourage him/her to try to find more words. If he/she is silent for 15 seconds repeat the basic instruction ('I want you to tell me all the animals you can think of'). No extension on the time limit is made in the event that the instruction has to be repeated.
- The score is the sum of acceptable animals. Any member of the animal kingdom, real or mythical is scored correct, except repetitions and proper nouns. Specifically each of the following gets credit: a species name and any accompanying breeds within the species; male, female and infant names within the species (max 40).

SCORE AS ½ POINT PER ANIMAL NAMED (ROUNDED).

TOTAL SCORE:

/15

WORD RECALL

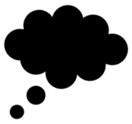

"A little while ago, I read you a list of words and you repeated the ones you could remember. Please tell me any of the words that you can remember now?"

- Allow up to one minute for recall.

SCORE AS 2 POINTS PER CORRECT WORD.

TOTAL SCORE:

/20

Word list option 1	tick	Word list option 2	tick	Word list option 3	tick	Word list option 4	tick
Hotel		Sky		Woman		Water	
River		Ocean		Rock		Church	
Tree		Flag		Blood		Doctor	
Skin		Dollar		Corner		Palace	
Gold		Wife		Shoes		Fire	
Market		Machine		Letter		Garden	
Paper		Home		Girl		Sea	
Child		Earth		House		Village	
King		College		Valley		Baby	
Book		Butter		Engine		Table	

Word Registration	Verbal Fluency	Word Recall	Total Score
/10	/15	/20	/45
