# Supplementary material for: Validation of a Harmonised, Three-Item Cognitive Screening Instrument for the Survey of Health, Ageing and Retirement in Europe (SHARE-Cog)
Source: Int J Environ Res Public Health. 2023 Sep 30;20(19):6869. doi: 10.3390/ijerph20196869 (PMC10572728; doi:10.3390/ijerph20196869)
Supplement: Supplementary file 1 [file ijerph-20-06869-s001.zip › Supplementary material.pdf]

Supplementary material

Table S1. Details on the age- and education- specific cut-offs for MCI and dementia that were obtained from the MoCA-based cognitive test battery.

Education level	Age group	Number (n=23,832)	Mean cognitive battery score	SD cognitive battery score	MCI cutoff (1 SD below the mean)	Dementia cut-off (2 SDs below the mean)	Values included (Dementia)	Percentage in this range	Values included (MCI)	Percentage in this range	Values included (SMC/NC)	Percentage in this range
Low	85+	1125	12.11	3.05	≤ 9.06	≤ 6.01	0–6	6%	7, 8, 9	15%	10–16	81%
Low	75-84	3417	13.24	2.52	≤ 10.72	≤ 8.2	0–8	6%	9, 10	8%	11–16	86%
Low	65-74	4106	14.15	2.05	≤ 12.1	≤ 10.04	0–10	6%	11, 12	11%	13–16	83%
Medium	85+	623	13.56	2.41	≤ 11.15	≤ 8.75	0–8	5%	9, 10, 11	10%	12–16	85%
Medium	75-84	3072	14.51	1.75	≤ 12.76	≤ 11.01	0–11	6%	12	9%	13–16	88%
Medium	65-74	5711	15.03	1.33	≤ 13.69	≤ 12.36	0–12	4%	13	7%	14, 15, 16	89%
High	85+	406	14.41	1.77	≤ 12.64	≤ 10.87	0–10	4%	11, 12	10%	13–16	86%
High	75-84	1878	14.99	1.44	≤ 13.55	≤ 12.11	0–12	6%	13	6%	14, 15, 16	88%
High	65-74	3494	15.33	1.14	≤ 14.19	≤ 13.05	0–13	6%	14	10%	15, 16	85%

Table S2. Details on those who were excluded from the analyses due to their cognitive diagnostic group being too unclear/contradictory including the numbers and rational.

Diagnostic category	Number of participants (primary analysis)	Memory disease diagnosis[1]	Objective deficit (cutoff range)[2]	IADL status (difficulties reported)[3]	SMC (Memory rated "fair" / "poor")[4]	Rational for inclusion/exclusion	Number of participants (Sensitivity analysis 1)	Number of participants (Sensitivity analysis 2)	Number of participants (Sensitivity analysis 3)
**Dementia**	62	Yes	Dementia	Yes	-	Met all criteria for dementia	-	84	81
Unclear/excluded	48	Yes	Dementia	No	-	IADL difficulties needed for dementia	-	26	29
**Dementia**	23	Yes	MCI	Yes	-	IADL difficulties suggests dementia	-	39	38
**MCI**	27	Yes	MCI	No	-	Met all criteria for MCI	-	11	12
**Dementia**	64	Yes	Normal	Yes	-	IADL difficulties suggests dementia	-	123	100
**MCI**	140	Yes	Normal	No	-	Disease diagnosis suggests MCI	-	81	104
**Dementia**	130	No	Dementia	Yes	Yes	Met all criteria for dementia	130	284	246
**Dementia**	56	No	Dementia	Yes	No	Met all criteria for dementia	56	159	128
Unclear/excluded	440	No	Dementia	No	Yes	IADLs difficulties needed for dementia	440	286	324
Unclear/excluded	591	No	Dementia	No	No	IADLs difficulties needed for dementia	591	488	519
Unclear/excluded	112	No	MCI	Yes	Yes	No IADL difficulties in MCI	112	286	238
Unclear/excluded	71	No	MCI	Yes	No	No IADL difficulties and SMC in MCI	71	251	186
**MCI**	611	No	MCI	No	Yes	Met all criteria for MCI	611	437	485
Unclear/excluded	1132	No	MCI	No	No	SMC needed for MCI	1132	952	1017
Unclear/excluded	370	No	Normal	Yes	Yes	No IADL difficulties in NC	370	1433	1033
Unclear/excluded	316	No	Normal	Yes	No	No IADL difficulties in NC	316	2083	1337
**SMC**	4957	No	Normal	No	Yes	Met all criteria for SMC	4957	3894	4294
**NC**	14,682	No	Normal	No	No	Met all criteria for NC	14,682	12,915	13,661
**Total participants**	20,752	-	-	-	-	-	20,436	18,027	19,149

Note Sensitivity analysis 2 and Sensitivity analysis 3 defined IADL status differently (details in Table S5).

[1] Self-reported doctor diagnosis of "dementia… or any other serious memory problem". The question also entails that they are being "treated for or bothered by" the condition.

[2] Age and education specific cut-off applied to a cognitive battert of tests. Dementia was defined as ≤ " SDs below the mean, MCI as between 1 SD and 2 SDs below the mean and normal ≥ 1 SD below the mean.

[3] Three instrumental activities of dailty living (IADLs) were consieded: tleephones calls, taking medications and managing finances.

[4] Those who reported a "serious memory impairment" diagnosis were considered to have a subjective memory complaint.

Table S3. Assessments of the impact of reducing maximum number of animals in the verbal fluency subtest on the overall model (registration, verbal fluency and recall) performance including the overall model fit measured using the pseudo R-squared (R_E^2) value and overall diagnostic accuracy of the model measured using the area under the ROC curve (AUC).

**Comparison**	**All 100 animals (R_E^2)**	**Max 60 animals (R_E^2)**	**Max 40 animals (R_E^2)**	**Max 30 animals (R_E^2)**	**Max 20 animals (R_E^2)**	**Max 10 animals (R_E^2)**
D_MCI_v_SMC_NC	0.140	0.140	0.140	0.141	0.143	0.134
(D+MCI) vs (NC)	0.199	0.199	0.199	0.199	0.200	0.186
MCI vs D	0.212	0.212	0.212	0.212	0.217	0.213
MCI vs (SMC+NC)	0.053	0.053	0.053	0.053	0.052	0.045
MCI vs NC	0.091	0.091	0.091	0.091	0.089	0.079
D vs (MCI+ SMC+NC)	0.211	0.211	0.211	0.211	0.214	0.203
**Comparison**	**All 100 animals (AUC)**	**Max 60 animals (AUC)**	**Max 40 animals (AUC)**	**Max 30 animals (AUC)**	**Max 20 animals (AUC)**	**Max 10 animals (AUC)**
D_MCI_v_SMC_NC	0.772	0.772	0.772	0.772	0.758	0.608
(D+MCI) vs (NC)	0.786	0.786	0.786	0.786	0.771	0.610
MCI vs D	0.740	0.740	0.740	0.740	0.739	0.682
MCI vs (SMC+NC)	0.726	0.726	0.726	0.726	0.710	0.556
MCI vs NC	0.743	0.743	0.743	0.743	0.725	0.558
D vs (MCI+ SMC+NC)	0.873	0.873	0.873	0.873	0.864	0.727

*Highest and lowest scores (at three decimal places) are marked in blue and red respectively.

Table S4. Diagnostic accuracy for all unique weighting combinations (n=291) obtained from varying the weightings of the three SHARE-Cog subtests (word registration, verbal fluency and word recall) between 0.5, 1, 2, 3, and 4 points per word and by changing the maximum number of animals in the verbal fluency subtest between 20, 30 and 40.

**Verbal fluency**	**Ratio of subtest scores**			**Total points**				**Diagnostic accuracy (AUC values)**						
**Maximum number of animals**	**Word registration**	**Verbal fluency**	**Word recall**	**Word registration**	**Verbal fluency**	**Word recall**	**Total score**	**(D+MCI) vs (SMC+NC)**	**(D+MCI) vs NC**	**MCI vs D**	**MCI vs (SMC+NC)**	**MCI vs NC**	**D vs (MCI+SMC+NC)**	**Average AUC value**

40	0.5	0.5	1.5	5	20	15	40	0.811	0.831	0.758	0.766	0.79	0.909	0.811
40	1	0.5	1.5	10	20	15	45	0.81	0.831	0.76	0.766	0.789	0.908	0.811
30	1	0.5	1.5	10	15	15	40	0.81	0.831	0.76	0.766	0.789	0.908	0.811
30	0.5	0.5	1.5	5	15	15	35	0.811	0.831	0.758	0.766	0.79	0.909	0.811
40	1	0.5	2	10	20	20	50	0.81	0.831	0.756	0.766	0.79	0.907	0.81
40	0.75	0.5	1	8	20	10	38	0.809	0.829	0.763	0.764	0.787	0.908	0.81
40	1.5	0.5	1.5	15	20	15	50	0.809	0.83	0.76	0.765	0.789	0.908	0.81
30	0.75	0.5	1	8	15	10	32	0.809	0.829	0.763	0.764	0.787	0.908	0.81
30	1	0.5	2	10	15	20	45	0.81	0.831	0.756	0.766	0.79	0.907	0.81
30	1.5	0.5	1.5	15	15	15	45	0.809	0.83	0.76	0.764	0.789	0.908	0.81
40	1.5	0.5	2	15	20	20	55	0.81	0.83	0.756	0.765	0.79	0.907	0.81
40	0.5	0.5	2	5	20	20	45	0.81	0.831	0.753	0.766	0.789	0.907	0.809
40	1.5	0.5	1	15	20	10	45	0.809	0.829	0.761	0.764	0.788	0.906	0.809
30	1.5	0.5	2	15	15	20	50	0.809	0.83	0.756	0.765	0.789	0.906	0.809
30	0.5	0.5	2	5	15	20	40	0.81	0.83	0.753	0.765	0.789	0.907	0.809
30	1	0.5	0.75	10	15	8	32	0.808	0.828	0.763	0.763	0.786	0.907	0.809
40	0.5	1	3	5	40	30	75	0.81	0.83	0.754	0.765	0.789	0.907	0.809
30	1.5	0.5	1	15	15	10	40	0.808	0.829	0.76	0.764	0.788	0.906	0.809
40	1	0.5	0.75	10	20	8	38	0.808	0.828	0.763	0.763	0.786	0.907	0.809
40	0.5	1	4	5	40	40	85	0.81	0.831	0.751	0.766	0.79	0.907	0.809
30	0.5	1	3	5	30	30	65	0.81	0.83	0.754	0.765	0.789	0.907	0.809
30	1	0.5	1	10	15	10	35	0.808	0.828	0.761	0.763	0.787	0.907	0.809
40	1	0.5	1	10	20	10	40	0.808	0.828	0.762	0.763	0.787	0.907	0.809
30	0.5	1	4	5	30	40	75	0.81	0.831	0.751	0.766	0.79	0.907	0.809
40	2	0.5	1.5	20	20	15	55	0.808	0.829	0.76	0.763	0.787	0.906	0.809
30	2	0.5	1.5	20	15	15	50	0.808	0.828	0.76	0.763	0.787	0.906	0.809
40	2	0.5	2	20	20	20	60	0.808	0.829	0.757	0.764	0.788	0.906	0.809
30	0.75	0.5	0.75	8	15	8	30	0.808	0.827	0.762	0.763	0.786	0.906	0.809
40	0.75	0.5	0.75	8	20	8	35	0.808	0.827	0.762	0.763	0.785	0.906	0.809
30	2	0.5	2	20	15	20	55	0.808	0.829	0.757	0.763	0.788	0.906	0.808
40	2	0.5	1	20	20	10	50	0.807	0.827	0.761	0.762	0.786	0.906	0.808

30	0.5	0.5	1	5	15	10	30	0.808	0.828	0.757	0.763	0.786	0.907	0.808
30	0.5	1	2	5	30	20	55	0.808	0.827	0.757	0.764	0.786	0.906	0.808
30	2	0.5	1	20	15	10	45	0.807	0.827	0.761	0.762	0.786	0.906	0.808
40	0.5	1	2	5	40	20	65	0.808	0.827	0.757	0.764	0.786	0.906	0.808
40	0.5	0.5	1	5	20	10	35	0.808	0.827	0.757	0.763	0.786	0.907	0.808
20	1	0.5	1.5	10	10	15	35	0.807	0.827	0.76	0.761	0.785	0.906	0.808
40	2	0.5	3	20	20	30	70	0.808	0.829	0.753	0.763	0.788	0.905	0.808
30	0.67	0.5	0.67	7	15	7	28	0.806	0.825	0.763	0.761	0.783	0.906	0.807
30	2	0.5	3	20	15	30	65	0.807	0.829	0.753	0.763	0.788	0.904	0.807
20	0.75	0.5	1	8	10	10	28	0.806	0.825	0.763	0.76	0.784	0.906	0.807
40	0.67	0.5	0.67	7	20	7	33	0.806	0.825	0.763	0.761	0.783	0.906	0.807
40	1	0.5	3	10	20	30	60	0.808	0.829	0.75	0.763	0.788	0.905	0.807
30	1	0.5	3	10	15	30	55	0.807	0.829	0.75	0.763	0.788	0.904	0.807
30	0.5	0.5	0.75	5	15	8	28	0.806	0.825	0.76	0.761	0.784	0.904	0.807
40	0.5	0.5	0.75	5	20	8	32	0.806	0.825	0.76	0.761	0.784	0.904	0.807
30	0.5	2	4	5	60	40	105	0.807	0.826	0.756	0.762	0.785	0.905	0.807
20	0.5	1	2	5	20	20	45	0.806	0.825	0.759	0.76	0.783	0.907	0.807
40	0.5	2	4	5	80	40	125	0.807	0.826	0.756	0.762	0.784	0.905	0.807
40	1.5	0.5	0.5	15	20	5	40	0.805	0.824	0.765	0.759	0.782	0.905	0.807
20	1	0.5	1	10	10	10	30	0.805	0.825	0.762	0.759	0.783	0.906	0.807
30	0.75	0.5	0.5	8	15	5	28	0.805	0.824	0.764	0.76	0.782	0.905	0.807
20	0.75	0.5	0.75	8	10	8	25	0.805	0.824	0.764	0.759	0.782	0.907	0.807
20	1	0.5	0.75	10	10	8	28	0.805	0.825	0.763	0.759	0.782	0.906	0.807
20	0.5	0.5	0.75	5	10	8	22	0.805	0.825	0.762	0.76	0.783	0.905	0.807
40	0.5	0.5	3	5	20	30	55	0.808	0.829	0.747	0.764	0.788	0.905	0.807
30	1.5	0.5	0.5	15	15	5	35	0.805	0.824	0.765	0.759	0.782	0.905	0.807
40	0.75	0.5	0.5	8	20	5	32	0.805	0.824	0.764	0.76	0.782	0.905	0.807
20	0.5	0.5	1.5	5	10	15	30	0.806	0.825	0.759	0.759	0.782	0.908	0.806
30	0.5	0.5	0.67	5	15	7	27	0.806	0.824	0.761	0.76	0.782	0.905	0.806
40	0.5	0.5	0.67	5	20	7	32	0.806	0.824	0.761	0.76	0.782	0.905	0.806
30	0.5	1	1.5	5	30	15	50	0.806	0.824	0.76	0.761	0.783	0.905	0.806

30	0.5	0.5	3	5	15	30	50	0.808	0.829	0.747	0.763	0.788	0.904	0.806
40	0.5	1	1.5	5	40	15	60	0.806	0.824	0.76	0.761	0.783	0.905	0.806
20	0.5	1	3	5	20	30	55	0.806	0.826	0.755	0.76	0.783	0.907	0.806
20	0.5	0.5	0.67	5	10	7	22	0.805	0.824	0.763	0.758	0.78	0.908	0.806
20	1	0.5	2	10	10	20	40	0.806	0.826	0.756	0.76	0.784	0.905	0.806
20	0.5	0.5	1	5	10	10	25	0.805	0.825	0.759	0.758	0.781	0.907	0.806
40	3	0.5	2	30	20	20	70	0.805	0.826	0.756	0.761	0.785	0.903	0.806
20	1.5	0.5	1.5	15	10	15	40	0.804	0.825	0.76	0.759	0.783	0.905	0.806
20	0.67	0.5	0.67	7	10	7	23	0.804	0.823	0.764	0.758	0.78	0.906	0.806
20	0.5	1	1.5	5	20	15	40	0.805	0.824	0.762	0.758	0.781	0.907	0.806
20	0.5	2	4	5	40	40	85	0.805	0.825	0.757	0.759	0.782	0.906	0.806
30	3	0.5	2	30	15	20	65	0.805	0.826	0.756	0.76	0.785	0.903	0.806
20	1.5	0.5	2	15	10	20	45	0.805	0.825	0.756	0.759	0.784	0.904	0.805
30	0.5	0.75	1	5	22	10	38	0.805	0.823	0.759	0.759	0.781	0.904	0.805
40	1	0.5	0.5	10	20	5	35	0.804	0.823	0.764	0.758	0.781	0.903	0.805
20	1.5	0.5	1	15	10	10	35	0.804	0.824	0.761	0.758	0.782	0.904	0.805
30	1	0.5	0.5	10	15	5	30	0.803	0.823	0.764	0.758	0.781	0.903	0.805
40	0.5	0.75	1	5	30	10	45	0.805	0.823	0.759	0.759	0.781	0.904	0.805
20	0.5	0.75	1	5	15	10	30	0.804	0.823	0.76	0.758	0.781	0.905	0.805
40	3	0.5	3	30	20	30	80	0.805	0.826	0.753	0.76	0.785	0.902	0.805
40	2	0.5	4	20	20	40	80	0.805	0.827	0.749	0.761	0.786	0.902	0.805
20	0.5	0.5	2	5	10	20	35	0.805	0.825	0.755	0.758	0.782	0.906	0.805
40	1	0.5	4	10	20	40	70	0.806	0.827	0.746	0.762	0.786	0.902	0.805
30	3	0.5	3	30	15	30	75	0.804	0.826	0.753	0.76	0.785	0.902	0.805
40	2	0.5	0.5	20	20	5	45	0.803	0.823	0.761	0.758	0.781	0.902	0.805
40	0.67	0.5	0.5	7	20	5	32	0.803	0.822	0.763	0.758	0.78	0.903	0.805
30	0.67	0.5	0.5	7	15	5	27	0.803	0.822	0.763	0.758	0.78	0.903	0.805
20	0.75	0.5	0.5	8	10	5	22	0.803	0.822	0.765	0.757	0.779	0.904	0.805
30	2	0.5	4	20	15	40	75	0.805	0.826	0.749	0.761	0.786	0.902	0.805
20	0.5	1	4	5	20	40	65	0.805	0.825	0.752	0.759	0.783	0.906	0.805
30	2	0.5	0.5	20	15	5	40	0.803	0.823	0.761	0.758	0.781	0.902	0.805

30	1	0.5	4	10	15	40	65	0.805	0.827	0.746	0.761	0.786	0.902	0.805
20	0.5	2	3	5	40	30	75	0.804	0.823	0.758	0.758	0.78	0.905	0.805
40	3	0.5	4	30	20	40	90	0.804	0.826	0.75	0.76	0.785	0.901	0.804
30	0.5	2	3	5	60	30	95	0.804	0.823	0.756	0.759	0.781	0.903	0.804
20	0.5	1.5	2	5	30	20	55	0.803	0.822	0.759	0.757	0.779	0.905	0.804
20	2	0.5	1.5	20	10	15	45	0.802	0.823	0.76	0.756	0.781	0.903	0.804
40	3	1	0.5	30	40	5	75	0.802	0.821	0.765	0.756	0.778	0.903	0.804
20	2	0.5	2	20	10	20	50	0.803	0.824	0.757	0.757	0.782	0.903	0.804
30	3	1	0.5	30	30	5	65	0.802	0.821	0.765	0.756	0.779	0.903	0.804
40	0.5	2	3	5	80	30	115	0.804	0.822	0.756	0.759	0.78	0.903	0.804
30	3	0.5	4	30	15	40	85	0.804	0.825	0.75	0.76	0.785	0.901	0.804
20	0.5	0.67	0.67	5	13	7	25	0.803	0.821	0.761	0.757	0.779	0.903	0.804
40	0.5	0.5	4	5	20	40	65	0.805	0.826	0.743	0.761	0.786	0.902	0.804
20	0.67	0.5	0.5	7	10	5	22	0.801	0.82	0.765	0.755	0.778	0.903	0.804
30	0.5	0.5	4	5	15	40	60	0.805	0.826	0.743	0.761	0.785	0.901	0.804
30	0.5	1.5	2	5	45	20	70	0.803	0.822	0.757	0.758	0.78	0.902	0.804
20	2	0.5	1	20	10	10	40	0.801	0.822	0.761	0.755	0.779	0.903	0.804
40	4	0.5	3	40	20	30	90	0.803	0.824	0.752	0.758	0.783	0.901	0.804
40	0.5	1.5	2	5	60	20	85	0.803	0.821	0.757	0.758	0.779	0.902	0.804
40	3	0.5	1	30	20	10	60	0.802	0.822	0.758	0.757	0.781	0.901	0.804
20	0.5	0.75	0.75	5	15	8	28	0.802	0.821	0.761	0.756	0.778	0.904	0.803
30	2	1	0.5	20	30	5	55	0.801	0.82	0.765	0.755	0.777	0.902	0.803
40	2	1	0.5	20	40	5	65	0.801	0.82	0.765	0.755	0.777	0.902	0.803
30	0.5	0.67	0.67	5	20	7	32	0.802	0.82	0.76	0.757	0.779	0.902	0.803
30	3	0.5	1	30	15	10	55	0.802	0.822	0.758	0.756	0.781	0.901	0.803
30	4	0.5	3	40	15	30	85	0.803	0.824	0.752	0.758	0.783	0.9	0.803
20	0.5	0.5	0.5	5	10	5	20	0.802	0.821	0.761	0.756	0.778	0.902	0.803
40	0.5	0.67	0.67	5	27	7	38	0.802	0.82	0.76	0.757	0.778	0.902	0.803
20	1.5	0.5	0.5	15	10	5	30	0.8	0.82	0.765	0.754	0.778	0.902	0.803
30	0.5	0.5	0.5	5	15	5	25	0.802	0.821	0.76	0.757	0.779	0.901	0.803
20	0.5	3	4	5	60	40	105	0.803	0.821	0.757	0.756	0.778	0.904	0.803

30	1	0.75	0.5	10	22	5	38	0.801	0.82	0.763	0.756	0.777	0.902	0.803
40	0.5	0.5	0.5	5	20	5	30	0.802	0.82	0.76	0.757	0.778	0.901	0.803
40	1	0.75	0.5	10	30	5	45	0.801	0.819	0.763	0.756	0.777	0.902	0.803
20	2	0.5	3	20	10	30	60	0.802	0.823	0.753	0.757	0.782	0.901	0.803
40	4	1	0.5	40	40	5	85	0.801	0.82	0.763	0.755	0.778	0.901	0.803
30	0.5	0.75	0.75	5	22	8	35	0.802	0.82	0.759	0.757	0.778	0.902	0.803
40	4	0.5	4	40	20	40	100	0.802	0.824	0.75	0.758	0.783	0.9	0.803
30	4	1	0.5	40	30	5	75	0.8	0.82	0.763	0.755	0.778	0.901	0.803
30	0.67	0.67	0.5	7	20	5	32	0.801	0.819	0.762	0.756	0.777	0.902	0.803
40	0.67	0.67	0.5	7	27	5	38	0.801	0.819	0.762	0.756	0.777	0.902	0.803
40	0.5	0.75	0.75	5	30	8	42	0.802	0.82	0.759	0.756	0.778	0.902	0.803
40	4	0.5	2	40	20	20	80	0.802	0.823	0.754	0.757	0.781	0.9	0.803
20	1	0.5	3	10	10	30	50	0.802	0.823	0.75	0.757	0.782	0.902	0.803
20	0.67	0.67	0.5	7	13	5	25	0.801	0.819	0.762	0.755	0.777	0.901	0.803
20	1	0.5	0.5	10	10	5	25	0.8	0.819	0.765	0.753	0.776	0.903	0.803
20	1	0.75	0.5	10	15	5	30	0.8	0.819	0.764	0.754	0.776	0.902	0.803
30	4	0.5	4	40	15	40	95	0.802	0.824	0.75	0.758	0.783	0.9	0.803
30	4	0.5	2	40	15	20	75	0.801	0.822	0.754	0.756	0.781	0.9	0.802
30	0.5	3	4	5	90	40	135	0.802	0.82	0.754	0.757	0.779	0.901	0.802
20	0.5	1	1	5	20	10	35	0.801	0.819	0.759	0.754	0.776	0.903	0.802
40	0.5	3	4	5	120	40	165	0.802	0.82	0.754	0.757	0.778	0.901	0.802
20	0.75	0.75	0.5	8	15	5	28	0.8	0.819	0.762	0.754	0.776	0.901	0.802
20	0.5	0.5	3	5	10	30	45	0.802	0.823	0.747	0.756	0.78	0.902	0.802
30	0.75	0.75	0.5	8	22	5	35	0.8	0.818	0.761	0.755	0.776	0.9	0.801
20	3	0.5	2	30	10	20	60	0.8	0.82	0.756	0.754	0.778	0.9	0.801
20	0.5	1.5	1.5	5	30	15	50	0.8	0.819	0.758	0.754	0.776	0.902	0.801
40	0.75	0.75	0.5	8	30	5	42	0.8	0.818	0.761	0.755	0.776	0.9	0.801
30	1.5	1	0.5	15	30	5	50	0.8	0.818	0.761	0.754	0.775	0.9	0.801
20	0.5	1	0.75	5	20	8	32	0.8	0.818	0.76	0.754	0.775	0.901	0.801
40	1.5	1	0.5	15	40	5	60	0.799	0.818	0.761	0.754	0.775	0.9	0.801
30	4	2	0.5	40	60	5	105	0.799	0.817	0.764	0.753	0.774	0.9	0.801

40	4	2	0.5	40	80	5	125	0.799	0.817	0.764	0.753	0.774	0.9	0.801
30	0.5	1	1	5	30	10	45	0.8	0.818	0.757	0.755	0.776	0.9	0.801
20	3	0.5	3	30	10	30	70	0.8	0.821	0.753	0.754	0.779	0.899	0.801
40	0.5	1	1	5	40	10	55	0.8	0.818	0.757	0.755	0.776	0.9	0.801
20	0.5	2	2	5	40	20	65	0.8	0.818	0.757	0.754	0.775	0.901	0.801
20	0.5	0.67	0.5	5	13	5	23	0.799	0.817	0.761	0.753	0.775	0.9	0.801
20	1.5	1	0.5	15	20	5	40	0.798	0.817	0.763	0.753	0.775	0.9	0.801
20	2	1	0.5	20	20	5	45	0.798	0.816	0.766	0.751	0.773	0.901	0.801
40	3	0.5	0.5	30	20	5	55	0.798	0.819	0.759	0.753	0.777	0.899	0.801
20	2	0.5	4	20	10	40	70	0.8	0.821	0.748	0.755	0.78	0.899	0.801
40	4	0.5	1	40	20	10	70	0.799	0.819	0.755	0.754	0.778	0.898	0.8
20	0.5	3	3	5	60	30	95	0.8	0.818	0.756	0.753	0.775	0.901	0.8
30	0.5	0.67	0.5	5	20	5	30	0.799	0.817	0.759	0.754	0.775	0.899	0.8
20	3	0.5	4	30	10	40	80	0.8	0.821	0.75	0.754	0.779	0.898	0.8
30	3	0.5	0.5	30	15	5	50	0.798	0.818	0.759	0.752	0.776	0.898	0.8
40	0.5	0.67	0.5	5	27	5	37	0.799	0.817	0.759	0.753	0.774	0.899	0.8
20	1	0.5	4	10	10	40	60	0.8	0.821	0.746	0.755	0.78	0.899	0.8
20	0.5	4	4	5	80	40	125	0.799	0.818	0.756	0.753	0.775	0.901	0.8
30	4	0.5	1	40	15	10	65	0.798	0.819	0.755	0.753	0.777	0.898	0.8
30	0.5	1.5	1.5	5	45	15	65	0.8	0.817	0.755	0.755	0.775	0.898	0.8
40	0.5	1.5	1.5	5	60	15	80	0.799	0.817	0.755	0.754	0.775	0.899	0.8
20	3	1	0.5	30	20	5	55	0.796	0.815	0.766	0.749	0.772	0.9	0.8
30	2	1.5	0.5	20	45	5	70	0.797	0.815	0.761	0.752	0.773	0.898	0.799
20	2	0.5	0.5	20	10	5	35	0.796	0.816	0.762	0.749	0.773	0.899	0.799
30	1	1	0.5	10	30	5	45	0.797	0.815	0.761	0.752	0.772	0.898	0.799
40	2	1.5	0.5	20	60	5	85	0.797	0.815	0.761	0.752	0.772	0.898	0.799
30	0.5	2	2	5	60	20	85	0.799	0.816	0.755	0.753	0.774	0.898	0.799
40	1	1	0.5	10	40	5	55	0.797	0.815	0.761	0.751	0.772	0.899	0.799
20	4	0.5	3	40	10	30	80	0.798	0.819	0.752	0.752	0.777	0.897	0.799
40	0.5	2	2	5	80	20	105	0.799	0.816	0.755	0.753	0.774	0.898	0.799
20	1	1	0.5	10	20	5	35	0.797	0.815	0.763	0.75	0.771	0.9	0.799

30	0.5	0.75	0.5	5	22	5	32	0.798	0.815	0.758	0.752	0.773	0.898	0.799
30	0.5	1	0.75	5	30	8	42	0.798	0.815	0.758	0.752	0.773	0.898	0.799
30	3	2	0.5	30	60	5	95	0.797	0.815	0.762	0.751	0.772	0.898	0.799
40	0.5	0.75	0.5	5	30	5	40	0.798	0.815	0.758	0.752	0.773	0.898	0.799
40	3	2	0.5	30	80	5	115	0.797	0.814	0.762	0.751	0.772	0.898	0.799
40	0.5	1	0.75	5	40	8	52	0.798	0.815	0.758	0.752	0.773	0.898	0.799
20	4	0.5	4	40	10	40	90	0.798	0.819	0.749	0.753	0.778	0.897	0.799
20	0.5	0.5	4	5	10	40	55	0.799	0.82	0.743	0.754	0.778	0.899	0.799
20	0.75	1	0.5	8	20	5	32	0.797	0.815	0.76	0.751	0.772	0.898	0.799
20	0.5	0.75	0.5	5	15	5	25	0.797	0.815	0.76	0.75	0.771	0.9	0.799
20	4	2	0.5	40	40	5	85	0.795	0.814	0.765	0.748	0.77	0.899	0.799
20	2	1.5	0.5	20	30	5	55	0.796	0.814	0.763	0.749	0.77	0.899	0.799
30	0.5	3	3	5	90	30	125	0.798	0.816	0.753	0.753	0.774	0.897	0.799
40	0.5	3	3	5	120	30	155	0.798	0.816	0.753	0.753	0.773	0.897	0.798
30	0.5	4	4	5	120	40	165	0.798	0.816	0.754	0.753	0.773	0.897	0.798
20	3	0.5	1	30	10	10	50	0.796	0.816	0.758	0.749	0.774	0.897	0.798
40	0.5	4	4	5	160	40	205	0.798	0.815	0.754	0.753	0.773	0.897	0.798
20	0.5	2	1.5	5	40	15	60	0.797	0.815	0.757	0.75	0.772	0.899	0.798
20	1.5	1.5	0.5	15	30	5	50	0.796	0.814	0.761	0.75	0.771	0.896	0.798
20	0.5	1.5	1	5	30	10	45	0.796	0.814	0.757	0.75	0.771	0.899	0.798
20	4	0.5	2	40	10	20	70	0.796	0.817	0.753	0.75	0.775	0.897	0.798
30	0.75	1	0.5	8	30	5	42	0.796	0.814	0.759	0.751	0.771	0.896	0.798
20	3	2	0.5	30	40	5	75	0.795	0.813	0.763	0.748	0.77	0.898	0.798
40	0.75	1	0.5	8	40	5	52	0.796	0.813	0.759	0.751	0.771	0.896	0.798
40	4	0.5	0.5	40	20	5	65	0.795	0.816	0.755	0.75	0.774	0.895	0.797
20	0.5	4	3	5	80	30	115	0.796	0.814	0.754	0.75	0.771	0.898	0.797
20	4	1	0.5	40	20	5	65	0.794	0.813	0.764	0.746	0.769	0.898	0.797
30	4	3	0.5	40	90	5	135	0.795	0.812	0.762	0.749	0.769	0.896	0.797
40	4	3	0.5	40	120	5	165	0.795	0.812	0.762	0.749	0.769	0.896	0.797
20	0.5	1	0.5	5	20	5	30	0.795	0.813	0.758	0.749	0.77	0.897	0.797
30	4	0.5	0.5	40	15	5	60	0.795	0.815	0.754	0.749	0.773	0.895	0.797

30	1.5	1.5	0.5	15	45	5	65	0.795	0.812	0.759	0.749	0.77	0.895	0.797
40	1.5	1.5	0.5	15	60	5	80	0.795	0.812	0.759	0.749	0.77	0.895	0.797
20	0.5	3	2	5	60	20	85	0.796	0.813	0.754	0.749	0.77	0.897	0.797
20	4	3	0.5	40	60	5	105	0.793	0.811	0.763	0.746	0.767	0.896	0.796
20	2	2	0.5	20	40	5	65	0.793	0.811	0.761	0.747	0.767	0.897	0.796
30	0.5	2	1.5	5	60	15	80	0.795	0.812	0.754	0.75	0.77	0.895	0.796
30	2	2	0.5	20	60	5	85	0.794	0.811	0.759	0.748	0.768	0.895	0.796
40	0.5	2	1.5	5	80	15	100	0.795	0.812	0.754	0.749	0.769	0.895	0.796
40	2	2	0.5	20	80	5	105	0.794	0.811	0.759	0.748	0.768	0.895	0.796
30	0.5	1.5	1	5	45	10	60	0.795	0.812	0.755	0.749	0.769	0.895	0.796
40	0.5	1.5	1	5	60	10	75	0.794	0.811	0.755	0.749	0.769	0.895	0.796
20	1.5	2	0.5	15	40	5	60	0.793	0.811	0.758	0.747	0.768	0.894	0.795
20	1	1.5	0.5	10	30	5	45	0.793	0.81	0.759	0.746	0.767	0.896	0.795
20	4	0.5	1	40	10	10	60	0.792	0.813	0.754	0.746	0.77	0.894	0.795
20	0.5	2	1	5	40	10	55	0.793	0.811	0.755	0.747	0.767	0.896	0.795
30	3	3	0.5	30	90	5	125	0.792	0.809	0.76	0.746	0.766	0.894	0.795
30	0.5	1	0.5	5	30	5	40	0.793	0.81	0.756	0.747	0.767	0.894	0.795
20	3	3	0.5	30	60	5	95	0.792	0.809	0.761	0.745	0.765	0.895	0.795
30	0.5	4	3	5	120	30	155	0.794	0.811	0.752	0.748	0.768	0.894	0.795
20	3	0.5	0.5	30	10	5	45	0.791	0.811	0.759	0.744	0.767	0.895	0.794
40	3	3	0.5	30	120	5	155	0.792	0.809	0.76	0.746	0.766	0.894	0.794
40	0.5	1	0.5	5	40	5	50	0.793	0.81	0.756	0.747	0.767	0.894	0.794
40	0.5	4	3	5	160	30	195	0.794	0.811	0.752	0.748	0.768	0.894	0.794
20	4	4	0.5	40	80	5	125	0.791	0.809	0.761	0.744	0.765	0.895	0.794
30	4	4	0.5	40	120	5	165	0.792	0.809	0.759	0.746	0.766	0.893	0.794
40	4	4	0.5	40	160	5	205	0.792	0.809	0.759	0.746	0.766	0.894	0.794
30	1	1.5	0.5	10	45	5	60	0.792	0.809	0.757	0.746	0.766	0.893	0.794
40	1	1.5	0.5	10	60	5	75	0.792	0.809	0.757	0.746	0.766	0.893	0.794
30	0.5	3	2	5	90	20	115	0.793	0.81	0.752	0.748	0.767	0.893	0.794
30	1.5	2	0.5	15	60	5	80	0.792	0.809	0.757	0.746	0.766	0.892	0.794
40	0.5	3	2	5	120	20	145	0.793	0.81	0.752	0.747	0.767	0.893	0.794

40	1.5	2	0.5	15	80	5	100	0.792	0.808	0.757	0.746	0.766	0.893	0.794
20	0.5	4	2	5	80	20	105	0.792	0.809	0.752	0.746	0.766	0.894	0.793
20	0.5	1.5	0.5	5	30	5	40	0.791	0.809	0.753	0.746	0.766	0.892	0.793
20	2	3	0.5	20	60	5	85	0.789	0.807	0.757	0.743	0.763	0.892	0.792
20	1	2	0.5	10	40	5	55	0.79	0.807	0.755	0.743	0.764	0.892	0.792
30	0.5	2	1	5	60	10	75	0.79	0.807	0.753	0.745	0.764	0.891	0.792
20	3	4	0.5	30	80	5	115	0.789	0.806	0.757	0.743	0.763	0.892	0.792
40	0.5	2	1	5	80	10	95	0.79	0.807	0.753	0.744	0.764	0.891	0.792
20	4	0.5	0.5	40	10	5	55	0.788	0.808	0.755	0.741	0.764	0.891	0.791
30	3	4	0.5	30	120	5	155	0.789	0.805	0.756	0.743	0.762	0.89	0.791
30	2	3	0.5	20	90	5	115	0.789	0.805	0.756	0.743	0.762	0.89	0.791
40	3	4	0.5	30	160	5	195	0.789	0.805	0.756	0.743	0.762	0.89	0.791
40	2	3	0.5	20	120	5	145	0.789	0.805	0.756	0.742	0.762	0.89	0.791
20	0.5	3	1	5	60	10	75	0.789	0.806	0.751	0.743	0.763	0.891	0.791
20	0.5	2	0.5	5	40	5	50	0.789	0.806	0.752	0.743	0.763	0.89	0.79
30	1	2	0.5	10	60	5	75	0.788	0.805	0.753	0.742	0.762	0.889	0.79
30	0.5	4	2	5	120	20	145	0.789	0.805	0.75	0.743	0.762	0.889	0.79
40	1	2	0.5	10	80	5	95	0.788	0.804	0.753	0.742	0.761	0.889	0.79
40	0.5	4	2	5	160	20	185	0.788	0.805	0.75	0.743	0.762	0.889	0.79
30	0.5	1.5	0.5	5	45	5	55	0.788	0.805	0.752	0.743	0.762	0.889	0.79
40	0.5	1.5	0.5	5	60	5	70	0.788	0.804	0.752	0.742	0.762	0.889	0.789
20	2	4	0.5	20	80	5	105	0.787	0.804	0.754	0.74	0.76	0.89	0.789
20	0.5	4	1	5	80	10	95	0.787	0.803	0.75	0.74	0.76	0.889	0.788
20	1	3	0.5	10	60	5	75	0.786	0.803	0.753	0.739	0.759	0.889	0.788
30	2	4	0.5	20	120	5	145	0.786	0.802	0.753	0.74	0.758	0.887	0.788
40	2	4	0.5	20	160	5	185	0.785	0.802	0.753	0.739	0.758	0.887	0.787
20	0.5	3	0.5	5	60	5	70	0.785	0.802	0.749	0.739	0.759	0.887	0.787
30	0.5	3	1	5	90	10	105	0.785	0.801	0.749	0.74	0.759	0.886	0.787
40	0.5	3	1	5	120	10	135	0.785	0.801	0.749	0.74	0.758	0.886	0.787
30	0.5	2	0.5	5	60	5	70	0.785	0.801	0.75	0.739	0.758	0.886	0.786
40	0.5	2	0.5	5	80	5	90	0.785	0.801	0.75	0.739	0.758	0.886	0.786

20	1	4	0.5	10	80	5	95	0.784	0.8	0.749	0.738	0.757	0.886	0.786
30	1	3	0.5	10	90	5	105	0.784	0.8	0.751	0.738	0.757	0.885	0.786
40	1	3	0.5	10	120	5	135	0.784	0.8	0.751	0.738	0.756	0.885	0.786
20	0.5	4	0.5	5	80	5	90	0.784	0.8	0.748	0.738	0.757	0.885	0.785
30	0.5	4	1	5	120	10	135	0.783	0.798	0.748	0.737	0.755	0.884	0.784
40	0.5	4	1	5	160	10	175	0.782	0.798	0.748	0.737	0.755	0.884	0.784
30	1	4	0.5	10	120	5	135	0.781	0.797	0.748	0.735	0.754	0.883	0.783
40	1	4	0.5	10	160	5	175	0.781	0.797	0.748	0.735	0.753	0.882	0.783
30	0.5	3	0.5	5	90	5	100	0.781	0.797	0.747	0.735	0.753	0.882	0.783
40	0.5	3	0.5	5	120	5	130	0.781	0.796	0.747	0.735	0.753	0.882	0.782
30	0.5	4	0.5	5	120	5	130	0.779	0.795	0.746	0.733	0.751	0.88	0.781
40	0.5	4	0.5	5	160	5	170	0.779	0.794	0.746	0.733	0.751	0.88	0.781

*Note for the analysis the subtests were weighted such that the lowest received 0.5 points per word and the total SHARE-Cog score was rounded to the nearest whole number. For presentation in the table above the scorings are ordered from the highest to the lowest average AUC value, are shaded by the total number of animals in the verbal fluency task and the chosen SHARE-Cog weighting is coloured red.

Table S5. Sensitivity Analysis of SHARE-Cog for different ways of defining the cognitive diagnostic groups.

Comparisons	Primary analysis	Sensitivity analysis 1	Sensitivity analysis 2	Sensitivity analysis 3
**Methods for defining cognitive categories**	**3 IADLs used:** Telephone; medication; money<br>**Positive memory disease diagnosis question used for MCI and dementia**	**3 IADLs used:** Telephone; medication; money<br>**Positive memory disease diagnosis question excluded for analysis 3**	**9 IADLs used:**<br>Telephone; medication; money;<br>Using a map; meal preparation;<br>Work house/garden<br>Doing laundry<br>Can leave house/travel independently<br>Grocery shopping	**9 IADLs used with additional qualifying criteria:**<br>Telephone; medication; money;<br>Using a map; meal preparation;<br>**Work house/garden [IF** No difficulty: stooping, kneeling, or crouching]<br>**Doing laundry [IF** No difficulty: stooping, kneeling, or crouching]<br>**Can leave house/travel independently [IF** No difficulty: walking 100m]<br>**Grocery shopping [IF** No difficulty: walking 100m AND No difficulty: Lifting or carrying weights over 10 pounds/5 kilos, like a heavy bag of groceries]
**Number by group**	**Numbers**	**Numbers***	**Numbers***	**Numbers***
Dementia	335	186	689	593

MCI	778	611	529	601
SC	4957	4957	3894	4294
NC	14682	14682	12915	13661
**AUC for SHARE-Cog**	**AUC (95% CI)**	**AUC (95% CI)**	**AUC (95% CI)**	**AUC (95% CI)**
D_MCI_v_SMC_NC	0.81 (0.8-0.82)	0.8 (0.79-0.82)	0.84 (0.82-0.85)	0.83 (0.82-0.84)
(D+MCI) vs (NC)	0.83 (0.82-0.84)	0.83 (0.81-0.84)	0.85 (0.84-0.86)	0.85 (0.83-0.86)
MCI vs D	0.76 (0.72-0.79)	0.76 (0.72-0.8)	0.71 (0.68-0.74)	0.73 (0.7-0.76)
MCI vs (SMC+NC)	0.77 (0.75-0.78)	0.77 (0.75-0.79)	0.77 (0.75-0.79)	0.76 (0.74-0.78)
MCI vs NC	0.79 (0.77-0.81)	0.79 (0.77-0.81)	0.79 (0.77-0.81)	0.78 (0.76-0.8)
D vs (MCI+ SMC+NC)	0.91 (0.89-0.92)	0.92 (0.9-0.94)	0.88 (0.87-0.9)	0.89 (0.88-0.9)

*Note the total number of particpants in the sensitivity anlyses are different since there will be a different number of people with unclear/excluded diagnostic categories (see Table S2 for further details).

Table S6. Results from the covariate-adjusted ROC curves of the SHARE-Cog including the adjusted area under the curve (AAUC), the regression coefficients (*p*-values) for each of the covariate levels and the R^2 value of the covariates combined.

Comparison	AAUC (95% CI)	(Intercept)	Age: 75-84	Age 85+	Education: medium	Education: high	Female sex	Countries*	R^2
(D+MCI) vs (SCI+NC)	0.80 (0.79-0.81)	19.60	-6.42 (<0.001)	-9.91 (<0.001)	3.68 (<0.001)	8.83 (<0.001)	0.70 (0.224)	(0 with p≤0.05)	0.283
(D+MCI) vs (NC)	0.82 (0.80-0.83)	19.60	-6.42 (<0.001)	-9.91 (<0.001)	3.68 (<0.001)	8.83 (<0.001)	0.70 (0.224)	(0 with p≤0.05)	0.283
MCI vs D	0.73 (0.69-0.77)	16.10	-3.62 (0.002)	-6.12 (<0.001)	1.52 (0.212)	6.03 (<0.001)	-1.23 (0.219)	(1 with p≤0.05)	0.202
MCI vs (SMC+NC)	0.76 (0.74-0.78)	21.17	-6.20 (<0.001)	-9.13 (<0.001)	3.99 (<0.001)	9.46 (<0.001)	1.47 (0.024)	(1 with p≤0.05)	0.333
MCI vs NC	0.78 (0.76-0.80)	21.17	-6.20 (<0.001)	-9.13 (<0.001)	3.99 (<0.001)	9.46 (<0.001)	1.47 (0.024)	(1 with p≤0.05)	0.333
D vs (MCI+ SMC+NC)	0.90 (0.88-0.92)	16.10	-3.62 (0.002)	-6.12 (<0.001)	1.52 (0.212)	6.03 (<0.001)	-1.23 (0.219)	(1 with p≤0.05)	0.202

*There were 19 countries included in total. Taking Austria as the reference country, none of the other 18 countries has a statistically sigificant impact on the diagnostic accuracy of the SHARE-Cog for (D+MCI) vs (SCI+NC) or (D+MCI) vs (NC) (i.e. all 18 *p*-values >0.05). Only Denmark -4.77 (p=0.029) [for MCI_v_D and D_v_MCI_SMC_NC] and Italy -3.16 (p=0.024) [for MCI_v_SMC_NC and MCI_v_NC] had marginally statistically significant results (at p≤0.05).
