# Supplementary figures and images for: Validation of a Harmonised, Three-Item Cognitive Screening Instrument for the Survey of Health, Ageing and Retirement in Europe (SHARE-Cog)
Source: Int J Environ Res Public Health. 2023 Sep 30;20(19):6869. doi: 10.3390/ijerph20196869 (PMC10572728; doi:10.3390/ijerph20196869)

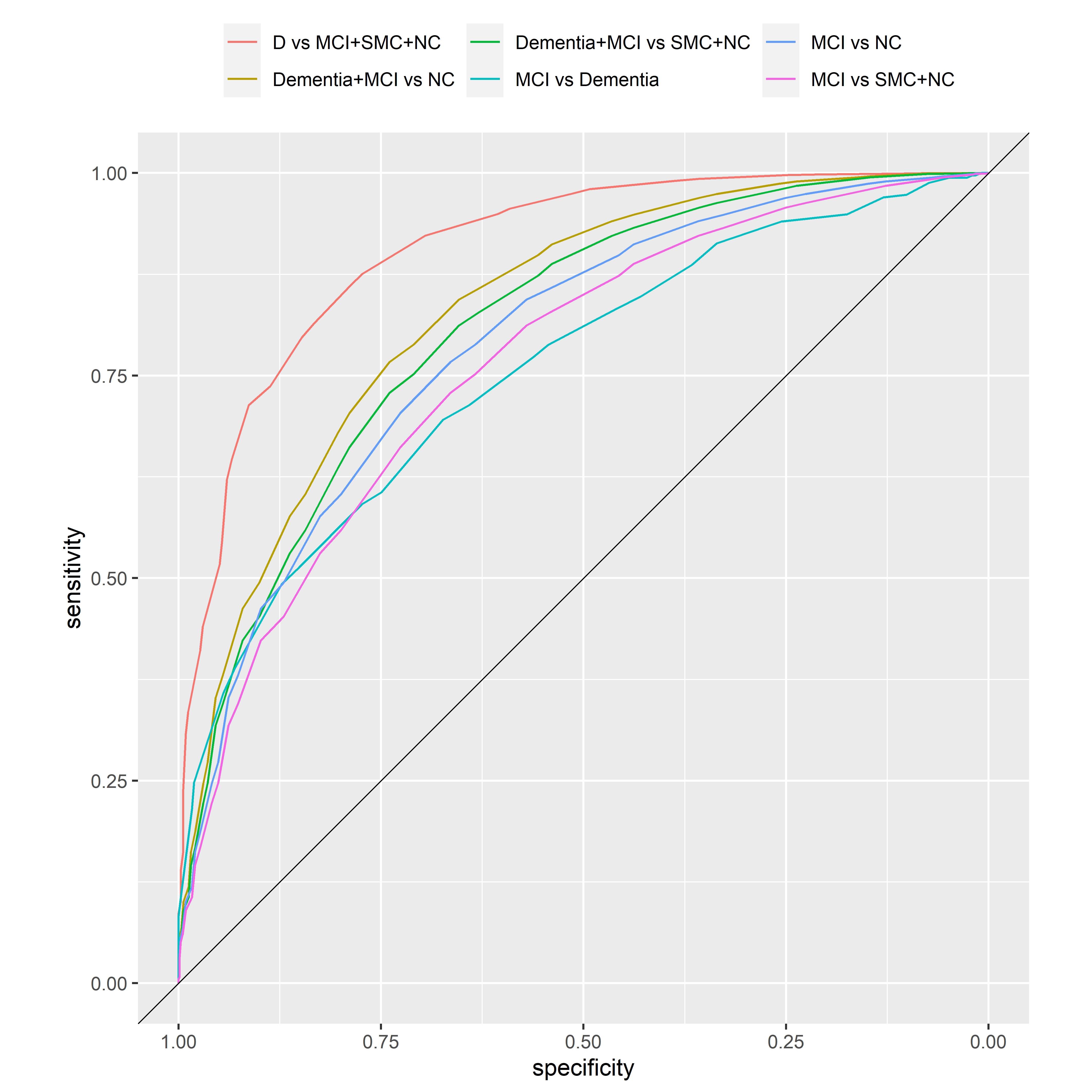

Supplement: Supplementary file 1 [file ijerph-20-06869-s001.zip › R_project_file/output/Figure3a.png]

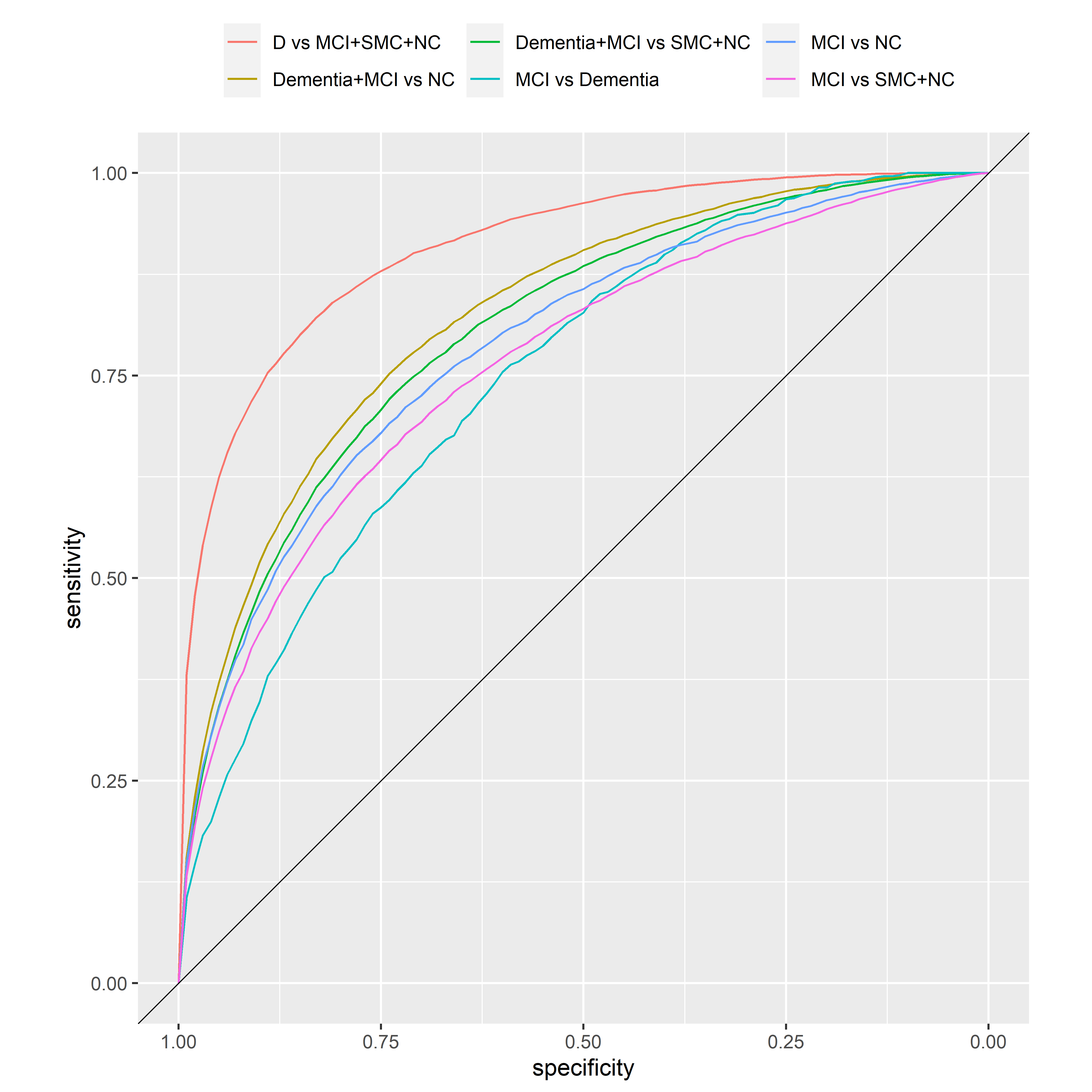

Supplement: Supplementary file 1 [file ijerph-20-06869-s001.zip › R_project_file/output/Figure3b.png]

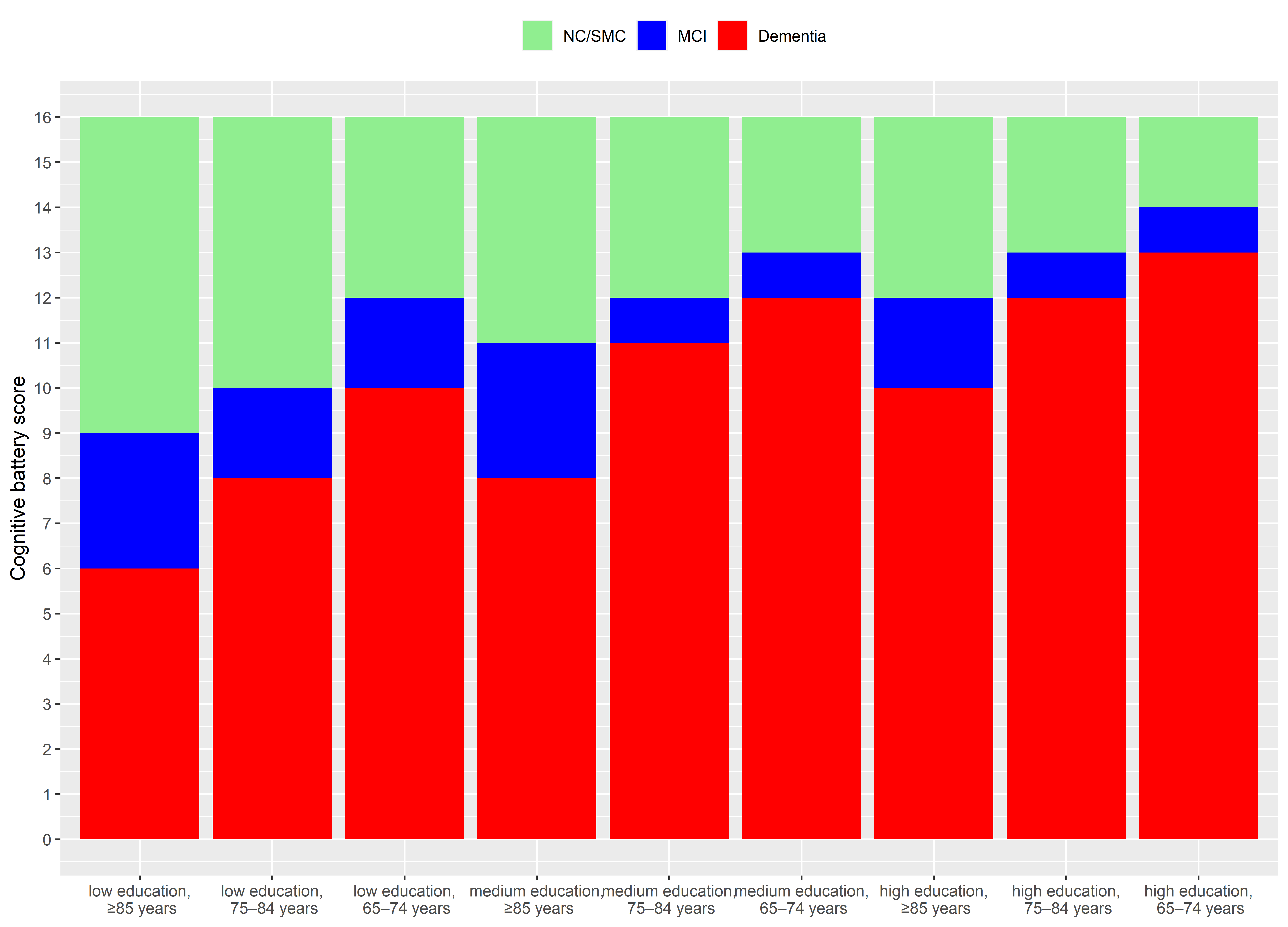

Supplement: Supplementary file 1 [file ijerph-20-06869-s001.zip › R_project_file/output/Figure_1.png]
